# Assessment of the Etiologic Factors of Gingival Recession in a Group of Patients in Northwest Iran

**DOI:** 10.5681/joddd.2009.023

**Published:** 2009-09-16

**Authors:** Ardeshir Lafzi, Nader Abolfazli, Amir Eskandari

**Affiliations:** ^1^Professor, Department of Periodontics, Faculty of Dentistry, Tabriz University of Medical Sciences, Tabriz, Iran; ^2^Assistant Professor, Department of Periodontics, Faculty of Dentistry, Tabriz University of Medical Sciences, Tabriz, Iran

**Keywords:** Case-control study, etiologic factor, gingival recession

## Abstract

**Background and aims:**

Gingival recession (GR), a common problem in periodontium, is associated with various etiologic factors. There is controversy over the role and importance of these factors. The aim of this study was to evaluate the etiologic factors of GR in a group of subjects in Northwest Iran.

**Materials and methods:**

In this case-control study, patients referring to a university clinic (123 patients with GR and 123 patients without GR) were evaluated. Patients were examined by an experienced periodontist. A checklist assessing the history of systemic disease, smoking, radiotherapy, orthodontic treatment, chemical and mechanical trauma, tooth-brushing method, type of occlusion, axial inclination of tooth, width and thickness of keratinized gingiva, presence of calculus, prosthesis, faulty restorations and food impaction, and frenum pull was completed for each patient. Chi-square test was used for data analysis.

**Results:**

Presence of calculus was significantly associated with GR in the evaluated patients (P = 0.000). Low width and thickness of keratinized gingiva, smoking and traumatic tooth brushing were other significant factors (P < 0.05). The type of occlusion, axial inclination of teeth, existence of prosthesis, high frenal attachment, radiotherapy, systemic diseases and chemical trauma were not significantly associated with GR in the evaluated patients (P > 0.05).

**Conclusion:**

Supra- and sub-gingival calculus, inadequate width and thickness of keratinized tissue, and incorrect tooth brushing techniques are most important etiologic factors of GR. Oral hygiene instructions including correct tooth brushing techniques as well as scaling and root planing with periodic recalls can play a significant role in prevention of GR.

## Introduction

Gingival recession is defined as an apical displacement of gingival margins to the cemento-enamel junction (CEJ), which results in root exposure.^1^ Gingival recession can be present in a normal periodontium or may be part of periodontal disease process.^2^

Epidemiologic studies reveal important information on prevalence and severity of a disease in a population and can be used to predict the disease pattern, progression, risk factors and treatment needs. Many studies have been conducted internationally on gingival recession. Study of National Health Center (NHC) of the United States from 1988 to 1991 among 7447 unemployed Americans revealed a 15% prevalence of gingival recession equal to or more than 3 mm. The prevalence varied from 0.5% in samples with 18 to 24 years of age to 45% in those over 65 years old.^3^

Gingival recession in patients with good oral hygiene appears as wedge-shaped lesion on buccal surface of the teeth, while in patients with poor oral hygiene, it can occur on any tooth surface.^4^ Following gingival recession, several complications like pain (hypersensitivity in cervical area of the teeth),^5^ probable tooth loss,^2^ loss of esthetic appperance,^6^ plaque retention,^2^ root caries (with a prevalence of 90% comparing to the 20-40% rate of prevalence in recession-free patients),^7^ and tooth abrasion may happen.^8^ Some of predisposing factors of gingival recession include:

- Bone anatomy: Gingival recession significantly increases in root surfaces which are not covered with bone (dehiscence).^9^
- Tooth position: Tooth eruption affects the amount of gingiva which surrounds the teeth^1^.
- Orthodontic movements could increase the probability of soft tissue recession.^10^
- Mechanical trauma: Frequent impaction of extrinsic objects leads to gingival recession.^2^ Furthermore, improper tooth brushing is an important factor for gingival recession in the areas with low plaque index.^11 - 13^
- Chemical trauma: Local cocaine use leading to gingival erosion is an example of chemical trauma.^14^
- High frenum attachment is attributed to local gingival recession.^15^
- Restorative dentistry: Subgingival restoration margins could increase plaque accumulation, gingival inflammation, bone resorption that lead to soft tissue recession.^16^
- Calculus: Several studies have shown calculus is an important factor in gingival recession.^1 , 4 , 6 , 17^
- Periodontal diseases result in connective tissue attachment loss and periodontal pocket formation or gingival recession.^18^
- Smoking: Several studies have demonstrated marginal recession is greater in smokers than non-smokers.^19 - 22^ Furthermore, smoking could adversely affect root coverage surgeries and decrease the success rate in smokers compared to non-smokers.^23^
- Removable prosthesis: Improper design of the removable dentures results in direct trauma and plaque retention, leading to gingival recession.^24^

The aim of the present study was to determine the etiologic factors of gingival recession in a group of patients in Northwest Iran.

## Materials and Methods

In this case-control study, 123 patients with gingival recession (26-60 years old; mean: 42.9) and 123 patients without gingival recession (26-60 years old; mean: 42.5) referring to the Department of Periodontics, Faculty of Dentistry, Tabriz University of Medical Sciences, Tabriz, Iran, were selected. After signing a written informed consent, a checklist was filled by an academic periodontist assessing the following criteria:

- Systemic diseases such as diabetes, hyperthyroidism and arthritis were evaluated based on medical history.
- Radiotherapy, assessed by medical history
- Orthodontic treatment, assessed by dental history
- Cigarette smoking, evaluated by pack-years (number of cigarettes smoked per day multiplied by the number of years that an individual smoked)
- Tooth brushing method and other mechanical traumas
- Chemical trauma, assessed according to history of chemical agents consumption like cocaine.
- Occlusion examination to find malocclusion, especially Class II div 2.
- Traumatic occlusion, assessed by increased mobility, widening of PDL in radiographs, vertical bone loss and infra-bony pockets and pathologic migration especially in anterior teeth.
- Deviation of tooth long axis, assessed by adjacent normal teeth and normal anatomy
- Presence of calculus, assessed by a periodontal probe and dentistry mirror.
- Evaluation of restorations (crown or Class V restoration), examined by tip of an explorer and dental mirror to detect the overhangs and recession around restorations
- Measurement of keratinized gingiva with periodontal probe.
- Frenum attachment was determined by tension of labial and buccal mucosa outward. If paling or displacement of free gingival adjacent to gingival recession area was observed, frenum was considered high-attached.
- Chi-square test was used for data analysis.

## Results

Supra- and sub-gingival calculus was significantly associated with gingival recession (P = 0.000). Also, inadequate width and thickness of keratinized gingiva as well as smoking were associated with gingival recession (P < 0.05).

From those with gingival recession, 18% did not brush their teeth at all, 42% brushed using scrub technique, and 40% used other improper techniques. Horizontal brushing (scrub technique) was significantly associated with gingival recession (P < 0.05). Other techniques including vertical and combination of horizontal and vertical tooth-brushing were not associated with gingival recession (P > 0.05). Different types of occlusion (Cl I, Cl II, and Cl III) could not be considered an etiologic factor in gingival recession (P > 0.05), although 3.8% of patients with gingival recession had deep bite. Trauma from tooth brushing and Class II div II was observed in 6.5% and 0.3% of patients with gingival recession, respectively, while no brushing or direct trauma was observed in recession-free patients.

Deviation of the long axis of teeth (inclination and rotation) was not attributed to gingival recession (P > 0.05). The presence of fixed or removable partial dentures did not prove to be an etiologic factor for gingival recession (P > 0.05), with only 0.8% of patients using such prostheses. Overhang restorations were observed in 6.5% of the patients with recession, but a significant association could not be established. Also, systemic diseases, radiotherapy, high-frenum attachment and chemical trauma were not associated with gingival recession (P > 0.05).

## Discussion

The aim of the present study was to determine the etiologic factors responsible for gingival recession in patients referring to the Tabriz University of Medical Sciences Faculty of Dentistry. The Department of Periodontics provides scaling/root-planing treatment as well as other periodontal therapies, and patients referring to this department could be considered a sample of the population in Tabriz, Northwest Iran.

Supra- and sub-gingival calculus were found to be the most important factor associated with gingival recession in the present study (P = 0.000). This finding is accordance with previous studies, highlighting calculus as the most important etiologic factor for gingival recession.^6 , 17^ Leo et al^4^ have also emphasized the role of poor oral hygiene, dental plaque and calculus in gingival recession. It is important to point out the reason for high prevalence of calculus in our study, which, according to the results, can be poor oral hygiene in individuals with gingival recession. Almost one-fifth of the studied patients did not brush their teeth at all, while others used improper tooth-brushing techniques. It is advisable to educate the patients to use proper tooth-brushing methods and other inter-dental aids such as interdental brushes for areas that brushing and flossing are less effective. This results in less plaque accumulation, less calculus formation and finally less periodontal disease and gingival recession. Cigarette smoking was another important etiologic factor in the studied population, which was significantly associated with gingival recession. This finding is in line with several previous studies.^19 - 22 , 25^

Brushing trauma was determined to be a significantly-associated etiologic factor of gingival recession, as seen in 42% of subjects using scrub technique. This is while none of the recession-free subject had brushing trauma. Trauma from tooth-brushing as an etiologic factor of gingival recession has also been noted in previous studies.^11 , 13 , 26^ In one study,^26^ an altered brushing technique was suggested for more than 90% of subjects who vigorously brushed their teeth resulting in gingival recession.

Width and thickness of keratinized gingiva was also an etiologic factor for gingival recession. However, studies show periodontal health can be maintained even in absence of attached gingiva.^27 , 28^ These studies have questioned the concept of a minimum required dimensions of keratinized gingival to maintain periodontal health. According to these findings, it could be assumed improper technique of tooth brushing is responsible for gingival recession in the evaluated group of patients in the present study.

Frenum attachment did not have a significant association with gingival recession in this study, contrary to the previous findings that show frenum attachment is an etiologic factor for gingival recession.^15^ The finding may be due to the small sample not yielding statistical significance. However, there is literature that shows frenum attachment tension occurs following gingival recession. In other words, frenum tension may be induced by gingival recession and not a causative factor of it.

A comprehensive study with a larger sample is suggested for determining the etiologic factors for gingival recession in the Iranian population.

The results of the present study emphasize on the importance of phase I therapy in preventing periodontal disease and gingival recession through reducing the accumulation of plaque and index.
